# Mercury Content in Impacted Wisdom Teeth from Patients of the Legnica–Głogów Copper Area—An In Vitro Pilot Study

**DOI:** 10.3390/jox13030029

**Published:** 2023-08-27

**Authors:** Sadri Rayad, Maciej Dobrzyński, Amadeusz Kuźniarski, Marzena Styczyńska, Dorota Diakowska, Tomasz Gedrange, Sylwia Klimas, Tomasz Gębarowski, Marzena Dominiak

**Affiliations:** 1 Academic Dental Polyclinic of Dental Center, Technology Transfer Ltd., Krakowska 26, 50-425 Wroclaw, Poland; 2 Department of Pediatric Dentistry and Preclinical Dentistry, Wroclaw Medical University, Krakowska 26, 50-425 Wroclaw, Poland; sylwia.klimas@umw.edu.pl; 3 Department of Prosthetic Dentistry, Wroclaw Medical University, Krakowska 26, 50-425 Wroclaw, Poland; amadeusz.kuzniarski@umw.edu.pl; 4 Department of Human Nutrition, Wroclaw University of Environmental and Life Sciences, Chelmonskiego 37/41, 51-630 Wroclaw, Poland; marzena.styczynska@upwr.edu.pl; 5 Department of Basic Sciences, Wroclaw Medical University, Chalubinskiego 3, 50-368 Wroclaw, Poland; dorota.diakowska@umw.edu.pl; 6 Department of Dental Surgery, Wroclaw Medical University, Krakowska 26, 50-425 Wroclaw, Poland; tomasz.gedrange@umw.edu.pl (T.G.); marzena.dominiak@umw.edu.pl (M.D.); 7 Department of Biostructure and Animal Physiology, Wrocław University of Environmental and Life Sciences, Kożuchowska 1/3, 51-631 Wroclaw, Poland; tomasz.gebarowski@upwr.edu.pl

**Keywords:** mercury, toxic metals, third molars, biomonitoring

## Abstract

The aim of this study was to determine the content of mercury in impacted third molars from Legnica–Głogów Copper Area residents to emphasize the effects of environmental pollution on the human body. A group of 72 patients with an average age of 27.3 ± 6.9 years participated in the study. Within this study, the research group (Legnica–Głogów Copper Area residents) comprised 51 individuals, while the control group (residents of Wrocław) consisted of 21 participants. A higher number of female individuals participated in the research (55). The amount of mercury present in the samples was determined through atomic absorption spectrometry with the use of a SpectraAA atomic absorption spectrometer and a V2 AA240FS flame attachment that utilized an air–acetylene flame. The accumulation of Hg in the teeth of members of the control group residing in Wrocław was studied, with a focus on identifying the risk factors that contribute to this phenomenon. The final model analyzed the presence of various factors, including thyroid and parathyroid gland diseases, cardiac diseases, and interval-scale Vit. D3 concentration. Among these factors, the presence of cardiac diseases was deemed statistically significant in relation to an increase in Hg concentration in third molars (rate ratio = 2.27, *p* < 0.0001). The concentration of mercury increased with the age and time of residence in the L-G Copper District.

## 1. Introduction

The conditions and changes in the environment have a huge impact on human health. They also influence the development and emergence of many diseases of civilization, including cancer. Biomonitoring is taking on greater importance in the context of health and the environment. The interests of environmental medicine focus on two aspects. The first covers the physiological, mental and emotional responses of the body to environmental factors. The second examines the causes of disease in an environmental context and develops methods for the detection, prevention, and control of diseases whose development is influenced by the target population’s environment. These aspects are very much linked to epidemiology. The combination of all factors gives an accurate picture of the health status of a region.

Knowledge of the health effects of environmental exposure provides a basis for specific health actions not only for individuals, but also for the population as a whole. The development of methods for detecting environment-related diseases is linked to the concept of biomonitoring. The concept of biomonitoring encompasses a range of actions aimed at evaluating the condition of the environment. These actions utilize bio-identifiers, which serve as indicators of pollution levels, in order to gauge the extent of environmental contamination [1].

In dentistry, a breakthrough occurred in the 1970s that resulted in an appreciation of the impact of external factors on the state of the oral cavity. In the 1970s and 1980s, an antibiotic called tetracycline was widely used, which showed similar staining properties on actively mineralizing tissues in both model animals and humans, enabling the determination of the dynamics of bone tissue synthesis. This was due to the formation of tetracycline–calcium–phosphate complexes, which had a brown color [2].

The oral cavity and the teeth are particularly vulnerable to metal accumulations. Apart from the metals present in the environment, metals are also utilized for the reconstruction of defects, for treatment, or for the filling of cavities within the oral cavity. According to the existing body of literature, there is compelling evidence to support the notion that mineralized tissues, such as teeth and bones, serve as an enduring testament to an individual’s way of life within a particular environment. This is due to the fact that these tissues effectively capture and retain the long-term effects of environmental contaminants [3]. Since their development has been well defined, they can serve as a basis on which to determine an organism’s exposure to toxic compounds (xenobiotics). This has enabled environmental monitoring using archeozoological and anthropological material, the biostructural analysis of human skeletons in forensic medicine, and animal experiments [4,5,6,7,8,9,10].

Environmental pollution by metals and their derivatives (compounds), and the resulting health risks, is an important area of interest for environmental medicine. Toxic metals (previously called heavy metals) can enter the human body by inhalation, by ingestion, or by absorption through the skin [11,12]. Metal and metal-alloy-based implants and prostheses have been utilized for over a century, and there have been reports of rejections, revisions, and toxicity being caused by metal particles. In recent years, concerns have grown regarding the complications arising from the use of metal ions, debris, and organo-metallic particles in orthopedic patients. Despite the well-established literature on environmental metal toxicants and safety limits for human exposure, efforts have not been sufficient in the case of implant-based metal toxicology. Serum metal ion concentration can serve as an indicator of systemic toxicity [13].

Typically, dental implants are constructed from alloys containing titanium. While this type of therapy for implants is currently perceived as having a positive outcome with no detrimental health effects, its success is contingent upon several different factors. To ensure the biocompatibility of implantable devices, it is crucial to discuss and define the characteristics of metals used for medical purposes. The presence of metals such as nickel, aluminum, and titanium in dental fillings, bridges, and implants can make them potential sensitizers. The benefits of endosseous prosthetics have garnered the attention of numerous dental professionals, leading to a rise in the implementation of implant-based procedures. With the prolonging of human life expectancy, there is a need for implant biomaterials that exhibit minimal adverse effects on the host’s tissues [14].

The movement of toxic metals throughout the ecosystem is primarily linked to the interconnectedness of plant–animal–human food chains [12,15,16]. Soil contamination with these substances is caused by industrial emissions, vehicular transportation, municipal waste management and their ingress from bedrock [16,17]. Among the most significant sources of emissions are the chemical, metallurgical, and mining industries, as well as soil fertilization and surface runoff from roadways with heavy vehicular traffic [12,18,19,20,21].

Toxic metals are absorbed by plants through water uptake from the soil and are subsequently stored within their tissues [22]. For most plants in our geographical area, these are mostly roots and leaves [23], readily eaten by animals and humans. Plants that strongly accumulate metals and their derivatives include, e.g., beetroot, potato, lettuce, and cabbage [24,25,26,27,28,29,30,31,32].

Thus, the most common route of entry of toxic metals into the body is the oral route; nevertheless, the extent of metal absorption is often influenced by various factors, including the pH level, the chemical composition the metals, the presence of other substances that might alter its absorption, and the rate at which it passes through the gastrointestinal tract. In contrast, the inhalation route allows the easiest absorption and distribution throughout the body via the circulatory system [12,33]. Skin appendages, such as glands or hair follicles, allow the incorporation of toxic metals, provided they are highly concentrated in the external environment [34].

The Legnica–Głogów Copper District is a highly developed and urbanized region, situated in the Province of Lower Silesia—specifically in its northern region. It is also a significant copper ore basin, spanning around 2.2 thousand square kilometers and accommodating approximately 500,000 residents. The district’s history dates back to the 1960s, during which the discovery of vast copper deposits spurred its growth. At present, the copper ore mining industry comprises three quarries: “Lubin”, “Rudna”, and “Polkowice-Sieroszowice”. This industry includes two essential components: ore enrichment plants and hydrotechnical plants. Additionally, the processing sector encompasses three distinct steelworks: “Legnica”, “Głogów”, and “Cedynia”. Lastly, the “Żelazny Most” engineering facility, which spans an area of 1400 hectares, serves as a storage site for copper ore tailings [35]. After analyzing the information provided, it can be inferred that the Legnica–Głogów Copper District would serve as an intriguing subject of study due to its significant issues with environmental contamination.

It is important to acknowledge that, in cases of brief and severe exposure, the quantity of poisonous substances that infiltrate the body is directly proportional to the degree of apparent impairment. In the case of prolonged moderate or weak exposure, the changes may be imperceptible, while non-specific disorders of organism functioning may appear, which are mostly not associated with toxic metal intoxication. The degree of toxicity of metals also depends on other factors, which include the chemical form in which they occur, their solubility in fats and in physiological fluids, the level of immunity of the organism concerned, and, finally, the duration of exposure [36,37].

An important factor modulating the toxic effects of metals is a person’s lifestyle and mass [38]. People with higher mass accumulate toxic metals more easily in soft tissues compared to slim people, in whom the same dose can lead to liver or kidney lesions. Conversely, people with high levels of adipose tissue, where more toxicity has accumulated, are at greater risk of the so-called reintoxification phenomenon, which occurs as a result of fat loss with age [39].

There are three primary classifications for the mechanisms by which metal toxicity occurs. The first is the obstruction of crucial functional groups found in proteins. The second is the displacement of metal ions that serve as cofactors for enzymes and other functional proteins. In addition, finally, the third classification involves the modification of the spatial configuration of proteins [40]. When metals bind to proteins, not only is the elimination of these metals from the body hindered, but their bioaccumulation levels are also increased [34]. This leads to the dysfunction of proteins in the cell. Such interactions can cause death upon exposure to high concentrations. When exposure levels do not exceed a lethal dose, poisoning manifests as structural, functional, or biochemical damage [36]. The most common electrodonor groups to which metal cations attach are the amine, carboxyl and thiol groups. Especially when attached to thiol groups, many enzymatic active centers are blocked. In addition, the use of antioxidative enzymes can serve as an early indicator of copper toxicity, even before the symptoms become visible [41].

Toxic metals tend to accumulate in specific organs, with the liver and kidneys being the primary centers for the detoxification and elimination of these metals. Additionally, other organs that are commonly affected include adipose tissue, muscle, nervous tissue, and bone [42,43,44]. Prolonged exposure to certain metals and their compounds can result in a gradual decline in physical, muscular, and neurological function that mimics the characteristics of illnesses like multiple sclerosis, Parkinson’s disease, Alzheimer’s disease, and muscular dystrophy. Furthermore, continued long-term exposure to these same metals and their compounds can even result in the development of cancer [12,45].

The movement of metals within the human body is somewhat restricted by biological barriers. However, when an excessive amount of toxic elements is present, the effectiveness of these barriers becomes limited. Metals can be categorized into different groups based on their varying levels of toxicity [46]:

- Minimal risk of harm: strontium (Sr), zirconium (Zr);
- Medium degree of potential harm: cobalt (Co), nickel (Ni);
- High degree of potential harm: iron (Fe), manganese (Mn), molybdenum (Mo),
- Exceptionally elevated degree of potential harm: zinc (Zn), chromium (Cr), cadmium (Cd), copper (Cu), lead (Pb), mercury (Hg).

**Figure 1:**
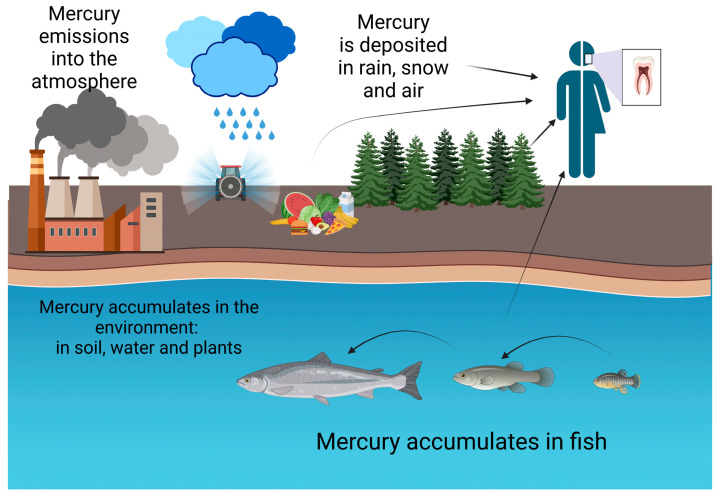
Sources of mercury.

The toxicity as well as the high levels of bioaccumulation of mercury have been well established. Mercury disrupts the tertiary and quaternary protein structure and modifies cellular functions by binding to selenohydryl and sulfhydryl groups, which react with methyl mercury and impair the cellular structure. Anthropogenic activities are the primary sources of mercury pollution, including agriculture, mining, municipal wastewater discharges, incineration, and industrial wastewater discharges (Figure 1). In nature, mercury exists in various forms, such as elemental or metallic forms, inorganic salts, and organomercurial compounds, each of which has different bioavailabilities and toxicities associated with them. The inhalation of mercury vapors can lead to bronchitis, asthma, and short-term respiratory issues. The following symptoms are associated with high-level exposure to metallic mercury: diarrhea, nausea, skin rashes, hypertension, and nephrotoxicity [12].

**Figure 2:**
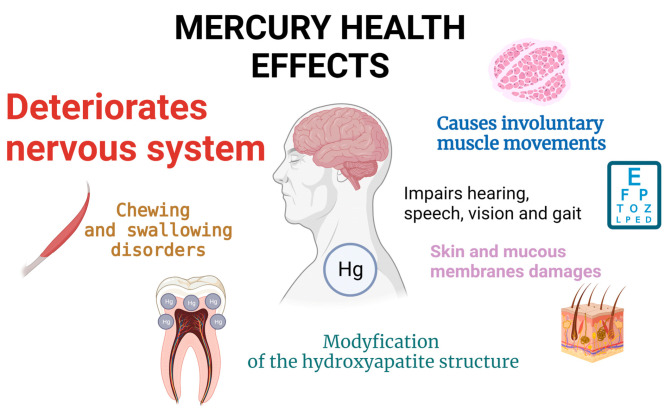
Mercury health effects.

An important point to consider is that mercury has the ability to accumulate in cartilage and bone tissue. During the development of molars, substitution occurs—mercury ions remain incorporated in the place of calcium ions in carbonates or hydroxyapatites [47] (Figure 2). In addition, there is intense metabolism of the developing bud, and an increased contact surface with the blood vessels, which can be used to transport Hg [48].

Vitamin D is a term that encompasses both vitamin D2 and D3. Vitamin D3 is produced by exposing lanolin’s 7-dehydrocholesterol to ultraviolet radiation. This results in the biological activity of cholecalciferol, which is also known as vitamin D3, and is naturally synthesized by the human skin [49]. However, it can also be obtained through dietary means, such as through foods or supplements. Unfortunately, not many natural sources of vitamin D exist, with the exception of certain oily fish, like mackerel, salmon, and herring. Vitamin D plays a significant role in the mineralization of teeth, and its levels have been garnering more attention in regard to oral health. During both growth and adulthood, a lack of vitamin D has been linked to various oral health issues. In children, a severe deficiency of vitamin D can lead to defects in mineralization, which can result in enamel and dentin defects. This could increase the risk of dental caries, and in adults, a lack of vitamin D has been connected with a higher prevalence of gingival inflammation and periodontitis [50,51].

In their study, Albawi et al. [52] investigated the occurrence of vitamin and mineral deficiencies, as well as excessive levels of non-essential heavy metals, among adults in Saudi Arabia. The incidence of vitamin D deficiency exhibited variations based on age. The prevalence of vitamin D deficiency was found to be higher in younger adults and men compared to older participants and women. Cho et al. [53] evaluated the relationship between blood mercury levels and osteoporosis in postmenopausal women. Elevated levels of Hg in the blood have been linked to a decreased risk of osteoporosis, as well as higher bone mineral density (BMD).

The aim of this study was to determine the amount of mercury found in the third molars removed from individuals living in the Legnica–Głogów Copper Area. This particular region poses an increased risk of heavy metal exposure due to its industrial and structural characteristics. The study also aimed to detect variations in mercury content in teeth among different groups of patients, as well as to evaluate the connection between the quantity of mercury found in the extracted third molars and the level of vitamin D detected in capillary blood.

## 2. Materials and Methods

### 2.1. Material

The authors were granted permission by the Bioethics Committee of Wrocław Medical University (consent number: KB-246/2019) before commencing this investigation. Following the principles outlined in the Declaration of Helsinki, the study was conducted on a cohort of 72 patients who had undergone the extraction of their third molars. These patients had provided informed consent for both the research and the medical procedure. Alongside the tooth extractions, blood samples were obtained and responses to a survey were collected. Patients were segregated into two groups—the research group had 51 patients, and the control group had 20 patients. The collection of research material took place between June 2020 and June 2021, coinciding with the COVID-19 pandemic, resulting in a limited number of patients.

For this study, the authors obtained lower impacted third molars from participants in both the control and study groups. The sample included the entire tooth’s extracted portion. To preserve the integrity of the samples, they were stored in aseptic containers at a temperature of −20 °C, without undergoing any chemical treatment, and were subsequently analyzed for mercury concentration.

Data regarding residency status and lack of dietary supplements as well as gender and age were gathered as part of the investigation. The patients had been living in either the Legnica–Głogów Copper Area or Wrocław since their birth, which was the primary inclusion criterion of the study. Additionally, the participants were in good health and had not taken any medication or supplements. Furthermore, capillary blood was collected for the determination of Vit. D3 level to appraise the correlation between the level of mercury and Vit. D3 concentration in patients residing in the L-G district.

### 2.2. Determination of Mercury in the Studied Materials

The mercury concentration was measured at the Department of Human Nutrition, Wrocław University of Environmental and Life Sciences. The dental material was subjected to multi-element analysis using atomic absorption spectrometry. The authors’ questionnaire yielded valuable data, encompassing a variety of personal details, including occupational exposure, history of allergies, age, gender, place of residence, smoking habits, general medical conditions, and any dietary supplements used by the individuals.

### 2.3. Mineralization of the Studied Materials

The process of mineralization of the samples occurred in a closed microwave system using a wet method. Homogeneous samples with weights ranging from 0.1 g to 0.5 g were taken, and 5 cm^3^ of nitric acid (V) A.C.S. along with 1 cm^3^ of concentrated hydrogen peroxide A.C.S. were added. The MARS 5 microwave sample preparation system was then used to mineralize the samples. Following mineralization, the minerals were transferred completely to measuring vessels with a capacity of 10 cm^3^, using redistilled water [54].

### 2.4. Examination of Mercury Content in the Samples

The estimation of mercury concentration in the samples was accomplished by atomic absorption spectrometry in an air–acetylene flame using a SpectraAA atomic absorption spectrometer; the flame was attached to a V2 AA240FS atomic absorption spectrometer, and dedicated cathode lamps were employed. The precision of the method was confirmed by utilizing the certified reference material—ERM-BD151 skimmed milk powder (Sigma-Aldrich, Saint Louis, MO, USA). This material has a measurement uncertainty of 5%. According to the specification provided by the manufacturer of the mercury analyzer, the limit of quantification (LOQ), defined as the smallest amount or lowest concentration of a substance that can be quantified using a given analytical procedure with the assumed accuracy and precision, is 0.0005 mg/kg for a sample of approximately 100 mg (0.05 ng Hg). The upper limit of the instrument range is 5 mg/kg, which for a 100 mg sample is equivalent to 500 ng Hg in the sample. Higher concentrations can be determined provided that the weight is reduced so as not to exceed 500 ng Hg per sample.

### 2.5. Methodology of Blood Collection and Determination of Vit. D3

The vitamin-D test system in the Vitality Health Check (VHC) was used to assess vitamin D levels from a capillary blood sample using the capillary method. To begin this process, the blood collection site was thoroughly cleansed, disinfected, and dried, and blood circulation was established. To obtain a blood sample, a lancet that was intended for a single use and was sterile was utilized to puncture the fingertip. Following the puncture, 10 µL of blood was collected with the aid of capillary tubes that had been treated with heparin. This collection process was facilitated by the use of a UniSampler blood collection device. The blood sample was then placed in the collection tube, which had previously been employed for buffer collection. After thorough mixing, the specimen was transferred to the test device using a micropipette. The VHC reader was used to read the vitamin D value 15 min later.

### 2.6. Statistical Analysis

Data analysis was conducted using the Statistica 13.3 software package (Tibco Software Inc., Palo Alto, CA, USA). The Shapiro–Wilk normality test was employed to evaluate data distribution. Descriptive data are presented as median (quartile 1–quartile 3, Q1–Q3). Chi-square test or Fisher’s exact test were utilized to analyze qualitative data. The non-parametric Mann–Whitney test was employed to compare quantitative data between two independent study groups. Techniques for the modeling of multivariable Poisson regression were used to identify the predictors of the Hg accumulation in third molars. A significance level of *p* ≤ 0.05 was adopted to determine statistical significance.

## 3. Results

### 3.1. Basic Characteristics of Patients

The demographic, clinical, and laboratory characteristics of both subgroups are presented in Table 1. There were 21 people in the control group. The control group consisted of 17 women and 4 men. Most patients in the control group were in the age group of 16–26 years (13 patients). There were a total of eight individuals whose ages fell within the span of 27 to 37 years. The absence of any statistical relevance regarding age within the control group could be attributed to the absence of a reference category. In the control group, no individuals were included with ages between 38 and 45. Only three people in the control group were smokers. The control group was primarily composed of individuals in white-collar professions, corresponding to a total of 15 patients. Of all the patients in the control group, only a solitary individual had heart disease. A meager two patients were found to have afflictions relating to their thyroid and parathyroid glands.

The data in Table 1 reveal that the majority of participants in the study group were young people between the ages of 16 and 26, who did not smoke and were generally in good health. In terms of gender distribution, age, smoking habits, occupation, illnesses, dietary supplements, and vitamin D3 levels, there were no significant distinctions between the study group and the control group (*p* > 0.05 for all variables). Additionally, there was a higher number of female participants than male ones. Notably, the extraction of teeth for surgical reasons was statistically significant (*p* = 0.003).

### 3.2. The Concentration of Mercury in the Extracted Teeth in Residents from the L-G Copper District and in the Control Group

The results presented in Table 2 indicate that patients from the L-G Copper District had slightly higher concentrations of Hg in their extracted teeth compared to the control group (0.389 and 0.341 (µg/g)); however, this was not statistically significant (*p*-value = 0.655).

### 3.3. The Risk Factors Contributing to the Build-Up of Mercury (Hg) in the Teeth of Individuals Living in the L-G District and in the Control Group

Multivariable Poisson regression revealed that the following factors constituted an optimal set of independent predictors of accumulation of Hg in people living in the L-G area: sex, age in interval scale, residence in the L-G Copper District, and reason for extraction. Table 3 provides a comprehensive overview of the primary factors that contribute to the build-up of mercury in the teeth of residents residing in the L-G district.

The risk of accumulation of Hg in the teeth was significantly decreased in younger subjects compared to in the oldest people (rate ratio = 0.77, *p* = 0.001). Decreased risk of the build-up of mercury (Hg) in the teeth was significantly related to shorter length of residence in the L-G area (rate ratio = −2.49, *p* < 0.0001 for time below 20 years and rate ratio = −0.64, *p* = 0.012 for time of residence 21–30 years). Increased risk of accumulation of Hg in third molars was significantly associated with times of residence in the L-G Copper District greater than 30 years (rate ratio = 3.14, *p* < 0.0001) and extraction for orthodontic reasons (rate ratio = 1.21, *p* < 0.0001) (Table 3).

We analyzed risk factors contributing to the build-up of Hg in the teeth of people in the control group, living in the Wrocław area, and the results are provided in Table 4. The following factors were analyzed in the final model: the presence of thyroid and parathyroid gland diseases, the presence of cardiac diseases, and Vit. D3 concentration in an interval scale. The presence of cardiac diseases was considered to be statistically significant for increased Hg concentration in third molars (rate ratio = 2.27, *p* < 0.0001). In contrast to the L-G Copper District group, there were no people aged 38–45 years old in the control group. This age range is a reference category for other age ranges in the multivariable Poisson regression, where the median concentration of Hg was 0.326 (0.285–0.493) µg/g for the 16–26-year-old subgroup vs. 0.346 (0.290–1.690) µg/g for 27–37-year-old subgroup. Therefore, no significant differences in concentrations of Hg were found in the control group in relation to age.

## 4. Discussion

The analysis of the aforementioned biological material for the presence of xenobiotics or their metabolites, as well as enzymes in inappropriate concentrations, makes it possible to assess the exposure of a given organism to harmful agents present in the environment [55]. The monitoring of xenobiotics, referring to toxic metals and persistent organic compounds (POPs), serves as the foundation for shaping environmental policies that impact the health of individuals residing in specific regions [56,57].

It is important to acknowledge that toxic metals are not biologically degradable. The process of detoxification involves either the concealment of active ions by metallothioneins or the deposition of these ions in insoluble forms, which are then either stored within the body or expelled from it afterwards [40,58]. Reducing the labile fraction of heavy metals in soil is crucial in managing plant toxicity, since it is the most significant factor. In order to mitigate the harmful effects of heavy metals present in contaminated soils, one effective chemical remediation method involves immobilizing the heavy metals in a form that is inaccessible to plants. This reduces their presence in the food chain, resulting in a lower risk of negative health and environmental impacts [59,60].

In addition, it is important to underscore that the form in which a metal is dissolved in lipids—i.e., whether it is a cation or an element—plays a significant role in determining which organs will be affected. For instance, when mercury is present in its cationic form, it tends to accumulate in the central or peripheral nervous system, while the elemental form has a strong affinity for the kidneys. Additionally, studies have shown that certain toxic metals have the ability to accumulate in specific organs. Lead tends to accumulate in bone tissues, while cadmium and mercury have a tendency to accumulate in parenchymal organs [61,62].

When present in high amounts, trace elements like copper, iron, manganese, and calcium can become hazardous, despite being necessary building blocks for cellular homeostasis. Dusek et al. [63] explored the manner in which discordance in metal balance leads to tissue accumulation and associated medical conditions. While certain disorders trigger the buildup of metals in specific areas of the brain, such as the globus pallidus, which is highly susceptible to divalent metal ion accumulation, other disorders generate widespread metal accumulation that can affect the whole brain, the liver, and other internal organs.

Certain elements that fall under the category of toxic metals are crucial for a variety of uses. Living organisms and elements with unknown physiological functions share a common trait: when consumed beyond their permissible limits, even those that are required in significant or trace quantities can have a harmful effect on all living organisms, including plants, animals, and humans. Even essential elements that are required in large or trace amounts can become hazardous once their threshold is surpassed. Among the most noxious metals are cadmium, mercury, and lead, which are not deemed necessary for living organisms. These metals have been known to cause a plethora of illnesses [13,16,45].

The assessment of the impact of chemical elements on living organisms in the environment has recently been approved as obligatory by law, and is known as biomonitoring. For human biomonitoring, non-invasive matrices like urine, saliva, and hair are the only options. Hair samples are ideal for evaluating prolonged exposure, as opposed to short-term exposure. The use of hair mineral analysis (HMA) has become an intriguing diagnostic tool for assessing toxic element exposure and evaluating health and nutritional status in the context of biomonitoring [64,65,66,67].

It is worth noting that, throughout the course of orthodontic treatment, the fixed appliance undergoes a variety of transformations. One such transformation occurs through changes in the surface of the appliance, which can lead to the emergence of metal elution or coverage on the surface due to the process of corrosion or passivation. Mikulewicz et al. [68] also examined the release of metal ions from orthodontic appliances and their sites of accumulation in pigs. The aorta, cheek, and hair sampled after three months showed the largest discrepancies in toxic metal content, with the experimental group exhibiting Ni levels 4.8 and 3.5 times higher, respectively, and Cr levels 3.4 times higher, respectively, than the control group.

The overall impact is greatly influenced by environmental conditions. Currently, the analysis of both dental health and overall physical well-being can be conducted using a combination of hair and tooth samples. The examination of bioindicators is a common practice for assessing environmental pollution levels. The examination of tooth and hair tissues can reveal the presence of different chemical elements. Shishniashvili et al. [69] evaluated primary teeth and hair as indicators of environmental pollution. Toxic elements were found to be at higher levels in polluted areas compared to areas with less pollution. The prevalence and severity of caries were found to be significantly related to regions with high levels of pollution. Furthermore, there was a clear correlation between the amount of pollution present and the extent of caries in affected populations.

Using atomic absorption spectrometry, Alomary et al. [70] conducted a study to measure the lead and cadmium levels in human teeth from Jordan. The results showed that the concentrations of Pb and Cd in the teeth of smokers were significantly higher than those of non-smokers. Meanwhile, Rayad et al. [71] examined the concentration of toxic metals, including Pb and Cd, in the third molars of people living in the Legnica–Głogów Copper Area. The study found that the content of all examined heavy metals increased with the age of the participants, but this was only found to be statistically significant for zinc, copper and chromium. Statistical analysis revealed that there were significantly higher levels of iron and lead in teeth extracted from patients residing in the L-G Copper Area than from those living in Wrocław, suggesting a possible link to increased environmental pollution in the former region during mineralization.

In a study conducted by Strugała-Stawik et al. [72], the average levels of lead in children’s blood were assessed, taking into account gender and proximity to the Legnica smelter. The study was conducted between 1991 and 2009. The findings of the research indicate a gradual decline in the concentration of lead in children’s blood. However, the presence of even small amounts of lead can have detrimental effects on cognitive abilities, learning aptitude, and memory retention, and have even been linked to the development of ADHD or antisocial behavior [73,74].

Mercury is categorized as a toxic metal, having earned the nickname “death metal” due to its extreme toxicity. Of all the potential sources of exposure to mercury, dental amalgam and mercury vapor from the production of chlorine are the most significant environmental sources, while occupational exposure is the most significant among all sources. Mercury dissolves in fats with ease, allowing it to infiltrate biological membranes. Mercury exposure has been linked with numerous harmful effects on various systems in the human body, such as the nervous system, the cardiovascular system, the endocrine system, and the kidneys. The detrimental impact of mercury on organ structure and function is well documented [75]. Mercury’s effects on the reproductive system should not be underestimated, as it can lead to endometrial dysfunction, implantation failure, premature delivery, subfertility, and spontaneous abortion [47,76].

Eide et al. [77] evaluated the content of mercury in primary teeth. Their research aimed to measure the amount of mercury present in primary teeth remaining from preindustrial times and compare it with a sample of primary teeth from modern-day Norway. According to the authors, the values obtained for mercury in the preindustrial primary teeth were indicative of minimal mercury levels being found in teeth, and were likely the result of natural environmental sources. Additionally, these values may serve as a benchmark for research on primary teeth from both preindustrial and contemporary time periods. Tvinnereim et al. [78] also analyzed the level of mercury in the primary teeth of humans, taking into account various factors that could influence the concentration of this metal. The research discovered variances in the concentrations of metal among the groups of teeth for lead, mercury, and zinc. Notably, positive correlations were found between lead and the other three metals, as well as between mercury and zinc.

Eide et al. [79] observed that rat teeth are prone to absorbing mercury once it has been introduced into the system, whether the mercury is in an organic or inorganic form. This finding is particularly significant, due to the prevalence of organic mercury in marine food sources. Conversely, inorganic mercury is the dominant form of mercury found in drinking water, and this can have significant ramifications in industrial contexts.

In the present study, it was found that the mercury content in extracted wisdom teeth was slightly higher in patients from the Legnica–Głogów Copper Area compared to patients from other areas (0.389 µg/g vs. 0.341 µg/g). However, this difference was not considered to be statistically significant (*p*-value = 0.655). It is possible that this may indicate an improvement in environmental conditions with respect to mercury. The lack of statistical significance with respect to age in the control group may be due to a missing reference category. There were no people aged 38–45 in the control group. Upon analysis of the data and the observations gathered, it was revealed that the concentration of mercury in the L-G Copper District escalated in conjunction with both age and duration of residency. In the control group, a noticeable correlation existed between the presence of cardiac ailments and the increase in Hg concentration.

Regular monitoring is deemed necessary, as anthropogenic pollution is found not only in industrialized and agricultural regions, but also in natural ecosystems, leading to the conclusion that it is widespread. In order to assess the extent of heavy metal pollution in the Warmia and Mazury region of Poland, Giżejewska et al. [80] analyzed the concentrations of mercury (Hg), lead (Pb) and cadmium (Cd) in the liver, muscles, and kidneys of European beavers (Castor fiber). The concentration of the elements was determined by means of atomic absorption spectrometry, and the metals were found to be present in all samples of individual tissues. Although the average concentrations of Pb and Hg were relatively low, the concentration of Cd was found to be high, particularly in the kidneys (7.933 mg/kg) and liver (0.880 mg/kg).

It is worth mentioning that dietary supplements containing shark liver oils, cod liver, and vegetables may potentially contain mercury. Brodziak-Dopierała et al. [81] examined mercury concentration in these supplements. The acceptable standard for the amount of Hg in dietary supplements (0.10 mg/kg) was not surpassed in any of the tested samples. In order to ensure consumer safety, it is also crucial to understand the levels of heavy metals present in crayfish tissues used for food due to the potential risk of contamination. Stanek et al. [82] conducted a study on the levels of mercury and other toxic metals detected in both the exoskeleton and abdominal muscles of spiny-cheek crayfish (Orconectes limosus) found in Lake Gopło. The heavy metal content in spiny-cheek muscles from Lake Gopło did not surpass the legal limits for fish and crayfish meant for human consumption, with the exception of lead.

In summary, biomonitoring enables comparative research on how environmental differences affect human health. This is accomplished by analyzing bone material taken from significant joint replacements or permanent teeth extracted for orthodontic or periodontal reasons. Teeth are a prime choice for biomaterials due to their accessibility and reliability as markers of exposure to environmental pollution. Furthermore, they provide a permanent, cumulative, and stable record of this exposure [70]. The third molars of individuals residing in the Legnica–Głogów Copper Area have proven to be a valuable biomonitor for determining environmental exposure to toxic metals.

## 5. Conclusions

The obtained results and observations showed that the concentration of mercury increased with the age and time of residence in the L-G Copper District. The presence of cardiac diseases was significantly associated with increased Hg concentration in the control group. Further investigation is needed to examine the influence of the habitat on the concentration of toxic metals in living organisms.

## 6. Study Limitations

The study was performed during the COVID-19 pandemic, between June 2020 and June 2021. Due to these circumstances, the number of patients in both groups was limited. Statistical tests were conducted, and test power was analyzed for both groups. After analyzing the concentration levels of Hg, we carried out a power examination that revealed the test power to be 0.65. The results indicated that the size of the study group was too small. While the authors acknowledge the limited scope of the study, they have plans to perform further examinations in future.

## Figures and Tables

**Table 1:** Demographic, clinical and laboratory characteristics of the total study group and the two subgroups of patients, divided according to place of residence. Descriptive data are presented as number of observation (percent) or median (Q1–Q3).

Variable	Total (n = 72)	L-G Copper District—No (n = 21)	L-G Copper District—Yes (n = 51)	*p*-Value
Gender:				0.558
male	17 (23.6)	4 (19.1)	13 (25.5)	
female	55 (76.4)	17 (80.9)	38 (74.5)	
Age (years old)	26.5 (16.0–45.0)	25.0 (24.0–27.0)	29.0 (22.0–32.0)	0.261
Age (years old):				0.080
16–26	36 (50.0)	13 (61.9)	23 (45.1)	
27–37	30 (41.7)	8 (38.1)	22 (43.1)	
38–45	6 (8.3)	0 (0.0)	6 (11.8)	
Smoking				0.590
no	64 (88.9)	18 (85.7)	46 (90.2)	
yes	8 (11.1)	3 (14.3)	5 (9.8)	
Occupation:				0.172
student	21 (29.2)	5 (23.8)	16 (31.4)	
worker	10 (13.9)	1 (4.8)	9 (17.6)	
white collar worker	41 (56.9)	15 (71.4)	26 (51.0)	
Years of living in the L-G Copper Area (n = 49):				1.000
≤20 years old	18 (35.3)	-	18 (35.3)	
21–30 years old	20 (39.2)	-	20 (39.2)	
≥31 years old	13 (25.5)	-	13 (25.5)	
Thyroid and parathyroid glands diseases:				0.971
no	65 (90.3)	19 (90.5)	46 (90.2)	
yes	7 (9.7)	2 (9.5)	5 (9.8)	
Cardiac diseases:				0.531
no	70 (97.2)	20 (95.2)	50 (98.0)	
yes	2 (2.8)	1 (4.8)	1 (2.0)	
Dietary supplements:				0.472
no	49 (68.1)	13 (61.9)	36 (70.6)	
yes	23 (31.9)	8 (38.1)	15 (29.4)	
Reason for extraction:				0.003 *
surgical	57 (79.2)	21 (100.0)	36 (70.6)	
orthodontic	8 (11.1)	0 (0.0)	8 (15.7)	
inflammation	7 (9.7)	0 (0.0)	7 (13.7)	
Vit. D3 (ng/mL)	27.0 (9.9–72.0)	27.8 (25.5–32.2)	26.4 (18.5–28.4)	0.134
Vit. D3 (ng/mL):				0.189
<20—large deficiency	19 (26.4)	3 (14.3)	16 (31.4)	
20–29—deficiency	36 (50.0)	10 (47.6)	26 (51.0)	
30–50—norm	11 (15.3)	6 (28.6)	5 (9.8)	
51–100—above the norm	6 (8.3)	2 (9.5)	4 (7.8)	

* Statistically significant.

**Table 2:** Tissue concentrations of Hg in the extracted teeth in the total study group and in both subgroups of patients, divided according to place of residence. Descriptive data are given as median (Q1–Q3).

Variable	Total (n = 72)	L-G Copper District—No (n = 21)	L-G Copper District—Yes (n = 51)	*p*-Value
Hg (µg/g)	0.367 (0.277–0.557)	0.341 (0.287–0.480)	0.389 (0.274–0.557)	0.655

**Table 3:** Poisson regression multivariable model of risk factors determining the accumulation of Hg in people living in the L-G area (n = 51).

Predictor	Risk Estimate	SE	95% Confidence Limits		Wald Test	*p*-Value
			Lower	Upper		
Intercept	3.64	0.87	1.92	5.35	17.33	<0.0001 *
Gender (for female)	−0.13	0.13	−0.39	0.14	0.96	0.325
Age (for 16–26 years old)	−0.45	0.32	−1.10	0.18	1.93	0.163
Age (for 27–37 years old)	0.77	0.23	0.31	1.23	10.67	0.001 *
Residence in the L-G Copper District (years)	−0.14	0.03	−0.21	−0.08	19.10	<0.0001 *
Residence in the L-G Copper District (for ≤20 years)	−2.49	0.56	−3.60	−1.38	19.32	<0.0001 *
Residence in the L-G Copper District (for 21–30 years)	−0.64	0.25	−1.15	−0.14	6.31	0.012 *
Residence in the L-G Copper District (for >30 years)	3.14	0.40	2.34	3.93	60.14	<0.0001 *
Reason for extraction (for orthodontic)	1.21	0.26	0.68	1.73	20.50	<0.0001 *

* Statistically significant.

**Table 4:** Poisson regression multivariable model of risk factors determining the accumulation of Hg in the control group (n = 21).

Predictor	Risk Estimate	SE	95% Confidence Limits		Wald Test	*p*-Value
			Lower	Upper		
Intercept	2.65	0.63	1.41	3.90	17.60	<0.0001
Thyroid and parathyroid glands diseases	0.71	0.41	−0.11	1.53	2.87	0.090
Cardiac diseases	2.27	0.32	1.62	2.91	47.35	<0.0001 *
Vit. D3 (for <20 ng/mL—large deficiency)	−0.60	0.73	−2.04	0.83	0.68	0.408
Vit. D3: (for 20–29 ng/mL—deficiency)	−0.57	0.56	−1.68	0.53	1.03	0.310
Vit. D3: (for 30–50 ng/mL—norm)	−0.81	0.61	−2.01	0.38	1.76	0.184

* Statistically significant.

## Data Availability

Not applicable.
